# *Toxoplasma gondii*, HBV, and HCV co‐infection and their correlation with CD4 cells among Iranian HIV‐positive patients

**DOI:** 10.1002/iid3.794

**Published:** 2023-02-23

**Authors:** Ahmadreza Bazmjoo, Mohammad Aref Bagherzadeh, Rahim Raoofi, Ali Taghipour, Samaneh Mazaherifar, Hojatallah Sotoodeh, Zahra Ostadi, Enayat Shadmand, Mirza A. M. Jahromi, Amir Abdoli

**Affiliations:** ^1^ Zoonoses Research Center Jahrom University of Medical Sciences Jahrom Iran; ^2^ Student Research Committee Jahrom University of Medical Sciences Jahrom Iran; ^3^ Department of Infectious Diseases Jahrom University of Medical Sciences Jahrom Iran; ^4^ Department of Parasitology and Mycology Jahrom University of Medical Sciences Jahrom Iran; ^5^ Department of Disease Control Fasa University of Medical Sciences Fasa Iran; ^6^ Department of Immunology Jahrom University of Medical Sciences Jahrom Iran; ^7^ Department of Advanced Medical Sciences & Technologies Jahrom University of Medical Sciences Jahrom Iran

**Keywords:** CD4, hepatitis B, hepatitis C, HIV/AIDS, Iran, T cell, toxoplasmosis

## Abstract

**Introduction:**

Human immunodeficiency virus (HIV/AIDS) infected patients have a higher risk of opportunistic infections (OIs) depending on their immunological status, especially CD4 + cell count. *Toxoplasma gondii*, hepatitis C virus (HCV), and hepatitis B virus (HBV) are important OIs among Human Immunodeficiency Virus (HIV)/Acquired Immune Deficiency Syndrome (AIDS) patients. However, little is known about co‐infection of these pathogens among HIV‐infected individuals and their correlation with the patient's CD4 + cell count. Hence, this study aimed to investigate the serological and molecular status of *T. gondii* infection among HIV‐infected individuals who had co‐infection with HBV and HCV infections.

**Methods:**

A total of 100 HIV/AIDS patients in two cities in the southwest of Iran was tested for *T. gondii* Immunoglobulin G (IgG) and Immunoglobulin M (IgM) antibodies as well as DNA detection by polymerase chain reaction (PCR) targeting the *RE* gene. HBV and HCV were detected by hepatitis B surface antigen (HBsAg) test, hepatitis C antibody (HCV Ab) test, and Real‐Time PCR. The number of CD4 + cell counts was determined by Flow cytometry.

**Results:**

Anti‐*T. gondii* IgG was positive in 22% of the patients, but anti‐*T. gondii* IgM and PCR were negative in all samples. HBV and HCV were positive in 8% and 33% of the patients, respectively. Co‐infections were as followed: HIV + HCV (16%), HIV + HCV + *T. gondii* (11%), HIV + *T. gondii* (5%), HIV + HBV (1%), HIV + HBV + *T. gondii* (1%), HIV + HBV + HCV (1%), and HIV + HBV + HCV + *T. gondii* (5%). A significant decline in CD4 + cell counts was found in such co‐infection groups (HIV + *T. gondii*, HIV + HCV + *T. gondii*, and HIV + HBV + HCV + *T. gondii*) compared with the HIV mono‐infection group.

**Conclusions:**

Our study showed that co‐infections of *T. gondii*, HCV, and HBV were common among HIV‐infected patients and co‐infections had a negative correlation with CD4 + cell counts of the patients.

## Graphical abstract

HIV/AIDS infected patients have a higher risk of opportunistic infections (OIs) depending on their immunological status, especially CD4 cell count. *Toxoplasma gondii*, hepatitis C virus (HCV), and hepatitis B virus (HBV) are important OIs among HIV/AIDS patients. The prevalence of HCV, HBV, and anti‐Toxoplasma IgG antibody were 33%, 8%, and 22%, respectively. Anti‐*T. gondii* IgM and PCR was negative in all samples. A significant decline in CD4 cell counts was found in such co‐infection groups (HIV + *T. gondii*, HIV + HCV + *T. gondii*, and HIV + HBV + HCV + *T. gondii*) compared with the HIV mono‐infection group. This study showed that co‐infections of *T. gondii*, HCV, and HBV had a negative correlation with CD4 counts of the patients.

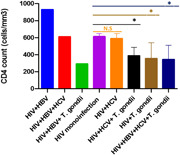

## 1 INTRODUCTION

The human immunodeficiency virus (HIV) is one of the major causes of morbidity and mortality around the world. According to the estimation, about 37.7 million people lived with HIV at the end of 2020. Accordingly, in 2020, 680,000 people died from HIV‐related causes and 1.5 million people acquired HIV.^1^ People living with HIV (PLWH) have a higher risk of opportunistic infections (OIs) dependent on their immunological status, stage of HIV, or adherence to antiretroviral therapy (ART).^2^, ^3^ Furthermore, CD4 T lymphocyte number has a critical role in disease progression and response to ART in PLWH,^4^, ^5^, ^6^, ^7^ while an increased incidence and number of OIs have been documented with diminishing CD4 + cell count.^2^, ^3^ As such, the clinical courses of OIs (e.g., toxoplasmosis and *Cytomegalovirus* infections) were correlated with a decline in the CD4 + cell count.^8^, ^9^

Toxoplasmosis, caused by the protozoan parasite *Toxoplasma gondii*, is an important OIs in HIV/AIDS patients.^3^, ^10^ The primary infection occurs through ingestion of contaminated water or food with oocysts shed by cats or by eating raw or undercooked meat containing tissue cysts of *T. gondii*.^11^, ^12^ Congenital transmission, blood transfusion, and organ transplantation are other important routes of *T. gondii* infection.^11^, ^12^ Toxoplasmosis is usually self‐limiting and asymptomatic in immunocompetent individuals,^12^ However, in immunocompromised patients, the infection could be life‐threatening (e.g., HIV/AIDS patients, organ transplant recipients, and patients with cancer) infection with fatal outcome.^13^, ^14^, ^15^, ^16^, ^17^ In HIV/AIDS patients, reactivation of latent toxoplasmosis may cause severe infections, which results in disseminated infection or encephalitis.^13^, ^18^, ^19^ According to a meta‐analysis, the pooled worldwide prevalence of *T. gondii*‐HIV co‐infection was estimated 35.8% (95% confidence interval [CI]: 30.8–40.7), while the prevalence in low‐income, middle‐income, and high‐income countries were 54.7% (95% CI: 35·8–73.5), 34.2% (95% CI: 27.4–40.9), and 26.3% (95% CI: 20.4–32.2), respectively.^10^ On the other hand, the number of CD4 + count (<200/mm^3^) is a significant risk factor for toxoplasmosis in PLWH.^20^ As such, the results of a recent meta‐analysis revealed that the risk of cerebral toxoplasmosis increases 27.94 times in PLWH with CD4 + T cells <100/mm^3^.^21^

Hepatitis B virus (HBV) and hepatitis C virus (HCV) are a major public health concern, especially among HIV infected patients.^22^, ^23^ As HIV, HBV, and HCV share similar transmission routes (e.g., sexual route, drug injection, and needle stick injury), co‐infection with these viruses is more common than in the general population.^23^, ^24^ Chronic hepatitis B (CHB) infection is a major cause of cirrhosis and hepatocellular carcinoma (HCC).^22^ According to a systematic review and meta‐analysis, the global prevalence of HBV among HIV individuals was 7.6% (5.6%–12.1%) or 2.7 (2.0–4.2) million co‐infections. Accordingly, the odds of HBV infection were 1.4 times higher among HIV‐positive compared to HIV‐negative individuals.^22^ HCV is a common opportunistic pathogen among HIV‐infected individuals, estimating a third of HIV‐infected individuals have HCV co‐infection in Europe and the United States of America (USA).^25^ HCV infection is a leading cause of chronic liver disease as well as liver failure and liver transplantation around the world.^23^, ^24^ In 2015, the worldwide prevalence of HIV‐HCV co‐infection was estimated 2,278,400 (1,271,300–4,417,000) cases, and the odds of HCV infection were six times higher in HIV‐infected individuals (5.8, 95% CI: 4.5–7.4) than their HIV‐negative counterparts.^26^ Furthermore, co‐infection of HIV with HCV and HBV accelerated liver injury^23^, ^24^, ^27^ and increased the risk of kidney disease.^28^, ^29^, ^30^ An inverse correlation between CD4 + cell count and persistent HBV viremia (CD4 < 200 mm^3^)^31^ and an increased risk of mortality (CD4 < 500/mm^3^)^32^ has been reported among PLWH coinfected with HBV. Progression of liver fibrosis was also correlated with declining CD4 + T cells among PLWH coinfected with HCV.^33^

Co‐infections can synergically augment the severity of some infectious diseases.^34^, ^35^, ^36^ For instance, virus‐virus co‐infection can heighten virus replication and persistence, altered immunological responses, and disease intensity.^37^ HIV‐HCV co‐infections promotes hepatocellular injury and boosts certain inflammatory cytokines.^38^ Our previous studies showed that maternal ToRCH (toxoplasmosis, rubella, CMV, and HSV) co‐infections were an increased risk of abortion among pregnant women than single infection.^39^ Accumulating evidence has shown that co‐infections have more severe consequences than the single infections.^35^

A number of studies reported a higher prevalence of toxoplasmosis in HIV‐infected individuals compared to healthy individuals.^10^, ^16^ However, little is known about the prevalence of triple co‐infection of *T. gondii*, HBV, and HCV and their correlation with CD4 + T cell count among PLWH. Hence, our study aimed to estimate the prevalence rates of *T. gondii*, HBV, and HCV infections among HIV‐infected individuals and their correlation with CD4 cell count.

## 2 MATERIALS AND METHODS

### 2.1 Study population and sampling

The present study was conducted among 100 confirmed cases of HIV‐infected individuals who had medical records in the health centers of Jahrom and Fasa cities (Fars Province, south of Iran) during 2020–2021. These cities have a hot semi‐arid climate, and each of them has more than 100,000 population. This study protocol was approved by the Ethical Committee of Jahrom University of Medical Sciences (IR.JUMS.REC.1398.065). The patient's information was extracted from their medical records. We obtained about five milliliters of venous blood samples from each patient, after centrifugation, the serum samples were separated for serologic evaluation, and the buffy coat samples were used for DNA extraction and molecular detection.

### 2.2 CD4 cell counts, HBV, and HCV status

Information about CD4 + T cell counts (determined by Flow cytometry), HBV, and HCV status were obtained from the patient's medical records. Accordingly, HBV and HCV were previously detected by hepatitis B surface antigen (HBs Ag) test, hepatitis C antibody (HCV Ab) test, and Real‐Time polymerase chain reaction (RT‐PCR).

### 2.3 Anti‐*T. gondii* antibody serologic test

Anti‐*Toxoplasma* IgM and IgG antibodies were detected by a commercial enzyme‐linked immunosorbent assay (ELISA) kit (Pishtaz Teb) according to the manufacturer's procedure. The cut‐off values at the upper and lower limit of 11 IU/mL were considered as positive and negative results, respectively.

### 2.4 Molecular detection of *T. gondii*

DNA was extracted from buffy coat samples using a commercial solution (DNG Plus) according to the manufacturer's protocol. The buffy coat samples were used for DNA extraction using the phenol–chloroform–isoamyl alcohol method, as described in a previous study.^40^ PCR was performed using a set of highly sensitive and specific primers for *T. gondii* (the *RE* gene) that amplified a region of 529 base pair (bp) fragments.^41^ The PCR primers^41^ and cycling conditions were described in previous reports (Tables S1 and S2).^42^ DNA of the *RH* strain of *T. gondii* was used as positive control and double distilled water was used as negative control. For each PCR reaction, a negative and positive control was included. PCR products were electrophoresed in agarose gel (stained with safe stain, Sinaclon, Iran) and visualized under UV transilluminator.

### 2.5 Statistical analysis

Correlations of CD4 + T cell counts with infection status were analyzed by statistics as a powerful statistical software SPSS (ver. 20) using Chi‐square test. The data are presented here as mean ± standard deviation (SD) of three independent experiments.

## 3 RESULTS

**Figure 1:**
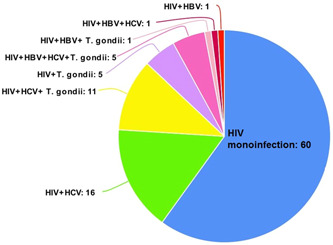
Pattern of co‐infection among HIV‐infected individuals. HBV, hepatitis B virus; HCV, hepatitis C virus; HIV, human immunodeficiency virus.

**Figure 2:**
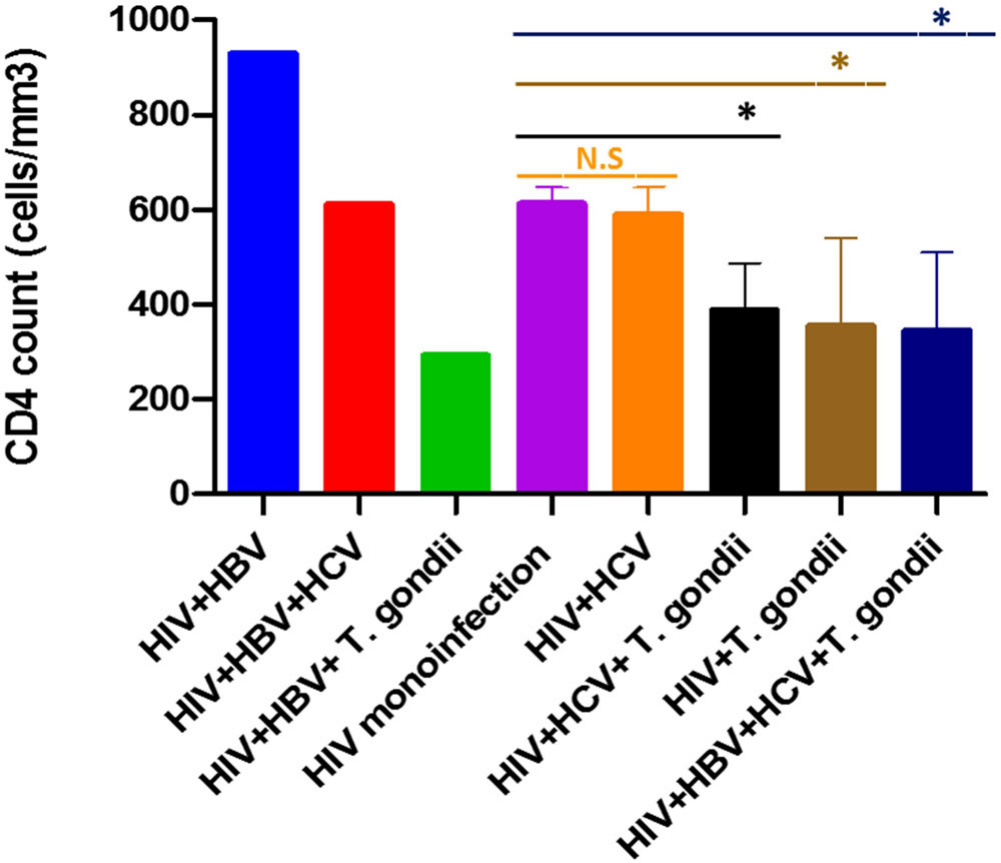
Correlation of CD4 cell counts with co‐infection status. Co‐infections were compared with the HIV‐monoinfection group (* *p* < .0001). We were unable to compare CD4 cell counts in HIV + HBV, HIV + HBV + HCV, and HIV + HBV + *T. gondii* groups because there was one patient in each of these groups. HBV, hepatitis B virus; HCV, hepatitis C virus; HIV, human immunodeficiency virus.

The mean ages of the patients were 43.79 years (±10.47 standard deviation (SD)) ranging from 20 to 63 years old. From the 100 patients, 52% and 48% were males and females, respectively (Table 1). HBV and HCV were positive in 8% and 33% of the HIV‐positive patients, respectively (Table 2). Anti‐*T. gondii* IgG was positive in 22% of the patients, but anti‐*T. gondii* IgM was negative in all samples. Moreover, *T. gondii* DNA was negative in all HIV‐positive patients (Table 2). Out of 100 HIV‐positive patients, 60% did not have co‐infection with HBV, HCV, or *T. gondii*, while the rest of the patients (40%) had co‐infections, including HIV + HCV (16%), HIV + *T. gondii* (5%), HIV + HBV (1%), HIV + HCV + *T. gondii* (11%), HIV + HBV + *T. gondii* (1%), HIV + HBV + HCV (1%), and HIV + HBV + HCV + *T. gondii* (5%) (Figure 1 and Table S3). There was a significant decline in CD4 + T cell counts in such co‐infection groups (HIV + *T. gondii*, HIV + HCV + *T. gondii*, and HIV + HBV + HCV + *T. gondii*) compared with the HIV mono‐infection group (Figure 2 and Table S3).

**Table 1:** Sex and age of HIV‐infected individuals.

Cities	Age, year (mean ± SD)	Sex	
		Male	Female
Jahrom (*N* = 50)	43 ± 9.89	29 (58%)	21 (42%)
Fasa (*N* = 50)	44.58 ± 11.07	23 (46%)	27 (54%)
Total (*N* = 100)	43.79 ± 10.47	52 (52%)	48 (48%)

Abbreviations: HIV, human immunodeficiency virus; SD, standard deviation.

**Table 2:** Infection status among HIV‐infected individuals.

Type of co‐infections	Jahrom			Fasa			Total		
	Male (*N* = 29)	Female (*N* = 21)	Total (*N* = 50)	Male (*N* = 23)	Female (*N* = 27)	Total (*N* = 50)	Male (*N* = 50)	Female (*N* = 50)	Total (*N* = 100)
HBV	2 (6.8%)	0	2 (4%)	4 (17.4%)	2 (7.4%)	6 (12%)	6 (12%)	2 (4%)	8 (8%)
HCV	7 (24.1%)	9 (42.8%)	16 (32%)	7 (30.4%)	10 (37%)	17 (34%)	14 (28%)	19 (38%)	33 (33%)
*Toxoplasma gondii*	6 (20.7%)	2 (9.5%)	8 (16%)	5 (21.7%)	9 (33.3%)	14 (28%)	11 (22%)	11 (22%)	22 (22%)

Abbreviations: HBV, hepatitis B virus; HCV, hepatitis C virus; HIV, human immunodeficiency virus.

## 4 DISCUSSION

In the current study, we performed sero‐molecular tests to screen *T. gondii* infection among PLWH. Overall, anti‐*T. gondii* IgG was positive in 22% of the patients, but anti‐*T. gondii* IgM and PCR was negative in all samples. Our findings revealed that out of 100 HIV‐positive patients, 60% did not have co‐infection with HBV, HCV, or *T. gondii*, while 40% of the patients had co‐infections. Of them, the prevalence of HCV, HBV, and *T. gondii* infection were 33%, 8%, and 22% among the HIV‐positive patients, respectively. Moreover, a significantly declined level of CD4 + T cell count was observed among *T. gondii* seropositive patients compared to the seronegative group. Generally, *T. gondii* IgG seropositivity without IgM and PCR positive results indicate latent infection. It should be noted that reactivation of latent toxoplasmosis is the major cause of toxoplasmic encephalitis (TE) in HIV/AIDS patients.^18^

Regarding *T. gondii*/HIV co‐infection in Iran, a previous study in Jahrom (the same region of this study) revealed that 21.1% of HIV‐infected individuals had anti‐*T. gondii* IgG antibody. Indeed, *T. gondii* seropositive patients had significantly lower levels of CD4 + T cell count than seronegative patients.^43^ Another report in western Iran revealed that anti‐*T. gondii* IgG and IgM seropositivity among 40.8% and 2.6% of HIV‐positive patients, respectively, although, no statistically significant correlation was found between toxoplasmosis and CD4 + T cell count.^44^ In the north of Iran, Rahimi et al.^45^ found a high seroprevalence rate of *T. gondii* IgG (96.3%) among HIV‐infected individuals, while IgM was negative in all of them. A recent study in the southwest of Iran demonstrated that 9.3%, 7.8%, and 9.3% of HIV‐positive patients had anti*‐T. gondii* IgG, IgM, and *T. gondii* DNA.^46^ A serologic study among HIV‐positive patients in Tehran (the capital of Iran) revealed that the prevalence rates of anti*‐T. gondii* IgG and IgM were 49.75% and 1%, respectively. It is worth noting a significant association was found between the rate of toxoplasma encephalitis and CD4 + T count (*p* < .001).^47^ A study among 208 HIV/AIDS patients In Shiraz, southern Iran, revealed that 18.2% of the patients had *T. gondii* seropositive, while TE was recorded in 89.6% and 10.4% of *Toxoplasma* seropositive and seronegative patients, respectively.^48^ The difference in seroprevalence rate of *T. gondii* among HIV‐infected individuals in Iran may be due to various factors, such as living in areas with humid climate, contact with cats and soil, consumption of raw/undercooked meat,^49^, ^50^ consumption of unwashed/raw vegetables.^51^ All of these factors could increase the exposure of human to the parasite. For example, the north of Iran (which has the highest seroprevalence rates of toxoplasmosis^46^) has a humid climate, which provides a suitable condition for parasite oocyst survival in the soil and environment.^52^ In this condition, percentages of latent infection among meat producing animals are more common than regions with low humid climate, leading to increased rates of meat‐born toxoplasmosis.^50^, ^53^, ^54^

We found that 40% of the patients had co‐infection (Figure 1 and Table S3). Interestingly, we found that the levels of CD4 + T cell counts were significantly declined in HIV‐positive patients who had co‐infections, including (HIV + *T. gondii*, HIV + HCV + *T. gondii*, and HIV + HBV + HCV + *T. gondii* compared with HIV mono‐infection group. A number of studies have been reported an increased prevalence rate of HCV^26^ and HBV^22^ among PLWH. Furthermore, studies suggest that co‐infection of HIV with HCV and HBV accelerates liver and kidney disease.^27^, ^29^, ^30^ On the other hand, there are evidence for an association between *T. gondii* infection and chronic liver disease (CLD)^55^, ^56^ as well as nonalcoholic fatty liver disease (NAFLD).^57^ A study in Burkina Faso demonstrated significantly higher prevalence rate of *T. gondii*/HBV, *T. gondii*/HCV, and HCV/HBV co‐infections among HIV‐positive pregnant women compared to HIV‐negative counterparts.^58^

A number of studies demonstrated that co‐infection can worsen the severity of infectious diseases.^34^, ^35^, ^59^ Virus‐virus co‐infection can enhance virus replication and persistence, dampen immunological response and increase disease intensity.^37^ HIV‐HCV co‐infection increased HCV RNA levels, promoted hepatocellular injury, inflammation, and fibrosis, and accelerates progression to cirrhosis and end‐stage liver disease.^38^, ^60^ Respiratory Syncytial Virus (RSV) exacerbated severity of influenza A virus disease in mouse models.^61^ ToRCH (toxoplasmosis, rubella, CMV, and HSV) co‐infections increased the risk of abortion than single infection among pregnant women.^39^ HIV and *T. gondii* co‐infection could disturb the immune regulatory mechanisms.^62^ El‐Sayed et al.^56^ found that a significantly increased parasitemia among CLD patients compared with the control group (30% vs. 6%; *p* < .001). Additionally, *T. gondii*/HBV and *T. gondii*/HCV co‐infection was 33.3% and 31.4%, respectively, alongside with a significant association between HCV viral load and *T. gondii* parasitemia. *T. gondii* positive CLD patients had a significant increase of liver enzymes than *T. gondii* negative patients.^56^ Accumulating evidence suggests that co‐infections have more severe sequels than the single infections.^35^

New evidence revealed that *T. gondii* can be sexually transmitted from male to female in humans^63^, ^64^, ^65^ as well as in animal models (*e.g*., Rats,^66^ dogs,^67^ sheep,^68^ and goats^69^). This is an important point because sexual transmission is one of the main routes of HIV, HBV, and HCV transmission.^23^, ^24^ Therefore, co‐infection of *T. gondii* with these viruses should be more considered for prophylaxis, screening, and management of the infection.

## 5 STUDY LIMITATIONS AND SUGGESTIONS

Our study has some limitations, including 1) limited sample size; 2) lack of clinical data of the patients; 3) lack of laboratory parameters of the patients. Based on these limitations, a larger investigation with a higher number of patients could be recommended in future investigations. Moreover, it should be recommended to obtain clinical data and laboratory findings of PLWH coinfected with toxoplasmosis, HBV, and HCV.

## 6 CONCLUSIONS

Our findings showed a high prevalence rate of co‐infection and their negative impact on CD4 + T cell counts of HIV‐infected patients. Routine screening of *T. gondii* as well as HCV and HBV should be recommended in HIV‐infected individuals.

## AUTHOR CONTRIBUTIONS

**Ahmadreza Bazmjoo**: Conceptualization; formal analysis; validation. **Mohammad Aref Bagherzadeh**: Formal analysis; methodology. **Rahim Raoofi**: Supervision. **Ali Taghipour**: Data curation; formal analysis; validation; visualization. **Samaneh Mazaherifar**: Methodology. **Hojatallah Sotoodeh**: Methodology. **Zahra Ostadi**: Methodology. **Enayat Shadmand**: Methodology. **Mirza Ali Mofazzal Jahromi**: Methodology; validation. **Amir Abdoli**: Conceptualization; funding acquisition; methodology; project administration; resources; supervision; writing—original draft; writing—review & editing.

## CONFLICT OF INTEREST STATEMENT

The authors declare that they have no conflicts of interest.

## ETHICS STATEMENT

This study was approved by the research and ethics committee of the Jahrom University of Medical Sciences, Jahrom, Iran (the ethics code: IR.JUMS.REC.1398.065).

## Supporting information

Supporting information.

## Data Availability

All data are available from the corresponding author upon reasonable request.
