# Supplementary material for: Toxoplasma gondii, HBV, and HCV co‐infection and their correlation with CD4 cells among Iranian HIV‐positive patients
Source: Immun Inflamm Dis. 2023 Feb 23;11(2):e794. doi: 10.1002/iid3.794 (PMC9947625; doi:10.1002/iid3.794)
Supplement: Supplementary file 1 — Supporting information. [file IID3-11-e794-s001.docx]

**Supplementary Table 1.** *T. gondii* and *N. caninum* specific primers targeting the *RE* and *Nc5* genes.

| **Primer name** | | **Sequences (5ʹ–3ʹ)** | **Expected size** | **Ref** |
| --- | --- | --- | --- | --- |
| ***T. gondii* RE primers** | | | | |
| PCR | TOX 4 | CGCTGCAGGGAGGAAGACGAAAGTTG | 529 bp | [1] |
|  | TOX 5 | CGCTGCAGACACAGTGCATCTGGATT |  |  |
|  | Np7 | GGGTGAACCGAGGGAGTTG |  |  |

1. Homan WL, Vercammen M, De Braekeleer J, Verschueren H. Identification of a 200- to 300-fold repetitive 529 bp DNA fragment in *Toxoplasma gondii*, and its use for diagnostic and quantitative PCR. Int J Parasitol. 2000;30(1):69-75. doi: <https://doi.org/10.1016/S0020-7519(99)00170-8>.

**Supplementary Table 2**. PCR conditions of the *RE* gene.

| **Reaction stages** | ***RE*** | |
| --- | --- | --- |
| **Initial denaturation** | 5 min, 94°C | 1 Cycle |
| **Denaturation** | 30 s, 94°C | 35 Cycles |
| **Annealing** | 30 s, **56**°C |  |
| **Extension** | 30 s, 72°C |  |
| **Final extension** | 10 min, 72°C | 1 Cycle |

**Supplementary table 3.** Correlation of CD4 cell counts with co-infection status.

| **Type of co-infections** | | **Number of co-infections** | **CD4 (Cell/mm^3^) (Mean±SD)** |
| --- | --- | --- | --- |
| 1 | HIV monoinfection | **N=60** (Jahrom: 29 cases and Fasa: 31 cases) | 612.18**±**285.83 |
| 2 | HIV+*T. gondii* | **N=5** (Jahrom: 4 cases and Fasa: one case) | 354.4**±**416.61 * |
| 3 | HIV+HCV | **N=16** (Jahrom: 13 cases and Fasa: 3 cases) | 589.37**±**240.29 |
| 4 | HIV+HBV | **N=1** (Fasa) | 929 † |
| 5 | HIV+HCV+ *T. gondii* | **N=11** (Jahrom: 2: cases and Fasa: 9 cases) | 387.18**±**331.05 * |
| 6 | HIV+HBV+ *T. gondii* | **N=1** (Jahrom) | 292 † |
| 7 | HIV+HBV+HCV | **N=1** (Fasa) | 610 † |
| 8 | HIV+HBV+HCV+*T. gondii* | **N=5** (Jahrom: one case and Fasa: 4 cases for) | 344.6**±**371.32 * |

* *P*<0.0001: HIV monoinfection *vs*. HIV+*T. gondii*

* *P*<0.0001 HIV monoinfection *vs*. HIV+HCV+ *T. gondii*

* *P*<0.0001 HIV monoinfection *vs*. HIV+HBV+HCV+*T. gondii*

*P* = 0.5420 HIV monoinfection *vs*. HIV+HCV

† Comparison between the groups with one patient were not possible.
